# MiR-184 expression is regulated by AMPK in pancreatic islets

**DOI:** 10.1096/fj.201701100R

**Published:** 2018-01-08

**Authors:** Aida Martinez-Sanchez, Marie-Sophie Nguyen-Tu, Ines Cebola, Arash Yavari, Piero Marchetti, Lorenzo Piemonti, Eelco de Koning, A. M. James Shapiro, Paul Johnson, Kei Sakamoto, David M. Smith, Isabelle Leclerc, Houman Ashrafian, Jorge Ferrer, Guy A. Rutter

**Affiliations:** * Section of Cell Biology and Functional Genomics Division of Diabetes, Endocrinology, and Metabolism, Department of Medicine, Imperial College London, London, United Kingdom;; † Beta Cell Genome Regulation Laboratory, Division of Diabetes, Endocrinology, and Metabolism, Department of Medicine, Imperial College London, London, United Kingdom;; ‡ Radcliffe Department of Medicine, University of Oxford, Oxford, United Kingdom;; § Department of Endocrinology and Metabolism, University of Pisa, Pisa, Italy;; ¶ San Raffaele Diabetes Research Institute (SR–DRI), Istituto di Ricovero e Cura a Carattere Scientifico (IRCCS) San Raffaele Scientific Institute, Milan, Italy;; ‖ Vita-Salute San Raffaele University, Milan, Italy;; # Hubrecht Institute, Utrecht, The Netherlands;; ** Department of Medicine, Leiden University Medical Center, Leiden, The Netherlands;; †† Clinical Islet Laboratory and Clinical Islet Transplant Program, University of Alberta, Edmonton, Alberta, Canada;; ‡‡ Nuffield Department of Surgical Sciences, University of Oxford, Oxford, United Kingdom;; §§ Nestle Institute of Health Sciences, Lausanne, Switzerland;; ¶¶ AstraZeneca Research and Development, Innovative Medicines and Early Development, Discovery Sciences, Cambridge, United Kingdom

**Keywords:** miRNAs, glucose, β cell, diabetes

## Abstract

AMPK is a critical energy sensor and target for widely used antidiabetic drugs. In β cells, elevated glucose concentrations lower AMPK activity, and the ablation of both catalytic subunits [β-cell–specific AMPK double-knockout (βAMPKdKO) mice] impairs insulin secretion *in vivo* and β-cell identity. MicroRNAs (miRNAs) are small RNAs that silence gene expression that are essential for pancreatic β-cell function and identity and altered in diabetes. Here, we have explored the miRNAs acting downstream of AMPK in mouse and human β cells. We identified 14 down-regulated and 9 up-regulated miRNAs in βAMPKdKO *vs.* control islets. Gene ontology analysis of targeted transcripts revealed enrichment in pathways important for β-cell function and identity. The most down-regulated miRNA was miR-184 (miR-184-3p), an important regulator of β-cell function and compensatory expansion that is controlled by glucose and reduced in diabetes. We demonstrate that AMPK is a potent regulator and an important mediator of the negative effects of glucose on miR-184 expression. Additionally, we reveal sexual dimorphism in miR-184 expression in mouse and human islets. Collectively, these data demonstrate that glucose-mediated changes in AMPK activity are central for the regulation of miR-184 and other miRNAs in islets and provide a link between energy status and gene expression in β cells.—Martinez-Sanchez, A., Nguyen-Tu, M.-S., Cebola, I., Yavari, A., Marchetti, P., Piemonti, L., de Koning, E., Shapiro, A. M. J., Johnson, P., Sakamoto, K., Smith, D. M., Leclerc, I., Ashrafian, H., Ferrer, J., Rutter, G. A. MiR-184 expression is regulated by AMPK in pancreatic islets.

Elevated blood glucose concentrations stimulate insulin secretion from the β cell but, in the long term, can lead to β-cell dysfunction and loss of β-cell identity, contributing to the development of type 2 diabetes (T2D) (1). AMPK is a fuel-sensitive enzyme involved in the control of whole-body glucose homeostasis and is a potential target for the treatment of T2D (2, 3). AMPK activation induces glucose uptake and utilization by peripheral tissues, especially skeletal muscle, and has been suggested to mediate the beneficial effects of exercise and of some antidiabetic drugs (2). In the β cell, AMPK is activated by glucose deprivation and may inhibit insulin secretion (4, 5). Recent studies (2, 3) suggest that chronic activation of AMPK may also impair insulin secretion.

Our group has recently generated two knockout models with β-cell–specific deletion of AMPK catalytic subunits α1 and α2 (βAMPKdKO, β-cell–specific AMPK double-knockout) or the main AMPK-upstream kinase liver kinase B1 (LKB1) (βLKB1KO, β-cell–specific LKB1 knockout) (6). βAMPKdKO and βLKB1KO islets showed increased glucose-stimulated insulin secretion *in vitro*, although gene inactivation only resulted in increased insulin secretion *in vivo* in the βLBK1KO animals. Moreover, both βAMPKdKO and βLKB1KO islets displayed a strong, but only partially overlapping, alteration of gene expression and thus impaired β-cell identity. Correspondingly, both βLKB1KO and βAMPKdKO islets displayed modified expression of several T2D-associated and T2D-regulated genes and increased expression of neuronal genes (6). These results suggest that chronic inhibition of AMPK might aggravate disease progression. However, the downstream targets and the underlying mechanism of AMPK action in the β cell remain largely unknown.

MicroRNAs (miRNAs) are 18–22 nt noncoding RNAs that play a critical role in the regulation of most biological processes and are implicated in the establishment and development of multiple diseases. miRNAs are generally transcribed by *Pol II* polymerase as longer primary transcripts that are quickly processed in the nucleus to generate ∼70–80 nt–long hairpin RNAs called premiRNAs. PremiRNAs are exported to the cytosol, where further processing generates the mature miRNAs that will induce translational silencing and/or degradation of the target mRNAs (7).

miRNAs are essential for the maturation of all endocrine populations (8, 9) and for mature β-cell function (10, 11). Specific miRNAs have been linked to processes associated with T2D, such as apoptosis, response to cytokines, or insulin secretion (12). Moreover, our work (11) and that of others (9, 10) supports the view that miRNAs are enforcers of β-cell identity (12, 13) and that their expression is altered in diabetes (12, 14). In response to insulin resistance, chronic increases in blood glucose levels are believed to contribute to the failure in β-cell function (15). Consistent with this model, glucose affects miRNA expression in β cells (12, 16). Other nutritional and environmental factors also alter the islet miRNome (12).

MiR-184 (miR-184-3p) has been identified as an important modulator of compensatory β-cell expansion during insulin resistance in the face of obesity (14, 17) and pregnancy (17) and is altered in diabetic mice and humans (14, 17). In mouse islets, miR-184 expression is down-regulated by glucose and palmitate *in vitro* (14, 18) and is strongly up-regulated by feeding with a ketogenic diet (18). Nevertheless, at present, the intracellular signaling pathways underlying the regulation of this and other β-cell miRNAs are largely unknown.

Here, we identified 23 miRNAs [Benjamini-Hochberg adjusted *P* value for false discovery rate (FDR) < 0.15] whose expression is altered in islets after β-cell–specific deletion of AMPK. Many of these miRNAs are predicted to control several processes important for β-cell function and identity. Moreover, we demonstrate that AMPK is a potent regulator of miR-184 expression in mouse and human islets and unveil a sexual dimorphism on miR-184 expression. Finally, we demonstrate that AMPK is required for glucose-dependent down-regulation of miR-184 *in vitro* and *in vivo*, providing a much sought-after link between the metabolic environment and miRNA expression in β cells.

## MATERIALS AND METHODS

### Generation and maintenance of transgenic mice

βAMPKdKO (AMPKα1^fl/fl^, AMPKα2^fl/fl^, Ins1-Cre^+/−^), control (AMPKα1^fl/fl^, AMPKα2^fl/fl^, Ins1-Cre^−/−^), βLKB1KO (LKB1^fl/fl^, Ins1-Cre^+/−^), control (LKB1^fl/fl^, Ins1-Cre^−/−^), and R299Q γ2 AMPK knock-in mice carrying 1 (heterozygous) or two (homozygous) mutant alleles were previously generated and maintained as described previously (3, 6) with free access to standard mouse chow diet or ketogenic diet (Ssniff, Soest, Germany). Glycemia was measured using tail vein blood and an AccuCheck Aviva glucometer (Roche, Basel, Switzerland). All *in vivo* procedures were approved by the UK Home Office Animal Scientific Procedures Act, 1986 (Licenses PPL 70/7349 and PA03F7F0F to I.L.).

### Isolation and maintenance of islets and cell lines

Mouse islets were isolated by digestion with collagenase as previously described (11) and, unless otherwise indicated, were allowed to recover from digestion overnight in culture medium [RPMI 1640, 10% fetal bovine serum (FBS), l-glutamine, and 11 mM glucose]. Human islets were maintained in RPMI 1640, 10% FBS, l-glutamine, and 5.5 mM glucose unless otherwise indicated. Donor characteristics of human islets are presented in **Table 1**. MIN6 cells were grown in high-glucose DMEM, 15% FBS, and l-glutamine.

**TABLE 1.** Donor characteristics of human islets as provided by the isolation center

Identifier	Body mass index (kg/m^2^)	Age	Origin facility
Male			
P49*^a^*	23.6	55	Edmonton, AB, Canada (McDonald)
P59	21.5	68	Pisa, Italy
P60	27.8	61	Milan, Italy
P66	31	52	Pisa, Italy
P74	24.5	83	Pisa, Italy
P76	22.9	75	Pisa, Italy
P80	35	54	Edmonton, AB, Canada (McDonald)
P81	29.7	65	Edmonton, AB, Canada (McDonald)
P84	NS	89	Pisa, Italy
P86	23	41	Leiden, The Netherlands
P89	24.5	64	Pisa, Italy
P94	29.4	51	Edmonton, AB, Canada (McDonald)
P97	28.7	62	Edmonton, AB, Canada (Shapiro)
P100	32.6	42	Pisa, Italy
P101*^a^*	35	57	Leiden, The Netherlands
P112	28.8	69	Edmonton, AB, Canada (McDonald)
P115	30.5	27	Edmonton, AB, Canada (Shapiro)
P124	22.9	18	Edmonton, AB, Canada (Shapiro)
P125	24.5	75	Pisa, Italy
P127*^a^*	32	57	Oxford, United Kingdom
P128	22.5	74	Pisa, Italy
P119	29.4	49	Edmonton, AB, Canada (Shapiro)
Female			
P58	21.7	68	Edmonton, AB, Canada (McDonald)
P77	33.1	66	Edmonton, AB, Canada (McDonald)
P78*^a^*	30.6	54	Edmonton, AB, Canada (McDonald)
P85	23.9	62	Pisa, Italy
P87	23	66	Pisa, Italy
P91*^a^*	21	53	Leiden, The Netherlands
P93	26	57	Milan, Italy
P102	31.5	78	Edmonton, AB, Canada (Shapiro)
P106	20.6	49	Milan, Italy
P113	32.3	30	Edmonton, AB, Canada (McDonald)
P114	35	46	Oxford, United Kingdom
P116	26	55	Milan, Italy
P117	24.5	52	Milan, Italy
P120	25.8	58	Pisa, Italy
P123	22	88	Pisa, Italy
P126	NS	NS	Edmonton, AB, Canada (McDonald)

^a^ Donors diagnosed with T2D.

AMPK activation was performed with 20 μM compound 13 (C13) (19) plus 50 μM compound 991 (C991) (20) in MIN6 cells or human islets for 14 or 24 h, respectively.

### RNA extraction, reverse transcription, and quantitative PCR

RNA, including miRNAs, was extracted using Trizol (Thermo Fisher Scientific, Waltham, MA, USA) following the manufacturer’s instructions.

MiRNA detection using quantitative PCR (qPCR) panels was conducted at Exiqon Services (Vedbaek, Denmark). Fifty nanograms RNA was reverse transcribed with the miRcury LNA Universal RT miRNA Kit (Exiqon). cDNA was assayed using the mouse and rat panel I + II and ExiLent SYBR Green master mix (Exiqon) in a LightCycler 480 System (Roche). Results were analyzed using Roche LC Software. Melting curves and melting temperature were confirmed for quality control, and only assays with 5 *C*_*q*_ less than the negative control and *C*_*q*_ <37 were included in the analysis. The average of assays detected in all samples was used as normalizer (average *C*_*q*_), determined by NormFinder (21) as the most stable normalizer [normalized *C*_*q*_ = average *C*_*q*_ − assay *C*_*q*_ (sample)]. Student’s *t* test was performed for each assay, and raw *P* values and *P* values adjusted for multiple testing by the Benjamini-Hochberg correction (*P*adj) are reported. Unsupervised hierarchical clustering and principal component analysis (PCA) were performed with the normalized Cq values. Fold change between control and βAMPKdKO islets was determined by the ∆∆*C_t_* method.

Individual miRNA RT-qPCR assays were performed as described above from 30 ng RNA in a 7900HT Fast Real-Time PCR System (Thermo Fisher Scientific). miR-7d-3p was identified as the miRNA with the least variability in the panel assays (using NormFinder) and was therefore chosen as the endogenous control.

RT-qPCR for protein-coding mRNAs and pri-miR-184 were performed as previously described (11). For pri-miR-184, the random primers were substituted by an anchored oligo dT primer (Thermo Fisher Scientific, Paisley, UK) in the reverse transcription reaction.

### Gene target and gene ontology analysis

TargetScanMouse (release 7.1) (22) was used to identify miRNA target genes. Tables containing prediction scores were merged with those containing gene fold change values in βAMPKdKO islets (6) using RStudio. Cytoscape (23) was used to visualize miRNAs and predicted targets. EnrichR (24) was used for gene ontology (GO) enrichment analysis, and REVIGO (25) was used to summarize and visualize enriched GO terms with *P*adj < 0.05.

### Immunoblot and immunohistochemistry

Slides were prepared from isolated pancreata and visualized as previously detailed (11). ImageJ software was used to calculate the mean intensity on phospho-AMPK Thr 172 in the β-cell area and in the acinar tissue surrounding the islets. Western blotting was performed as previously described (11) with 100–150 human or mouse islets. Anti–acetyl-CoA-carboxylase (ACC), anti-phospho-ACC, anti-AMPK, anti–phospho-AMPK Thr 172, anti-Raptor, and anti–phospho-Raptor were from Cell Signaling Technology (Danvers, MA, USA). Anti-glucagon was from Sigma-Aldrich (St. Louis, MO, USA).

### Statistical analysis

Statistical significance was evaluated with GraphPad Prism 7.0 software (GraphPad, La Jolla, CA, USA) as indicated in the figure legends. All data are shown as means ± sem. Unless otherwise indicated, *P* < 0.05 was considered statistically significant.

## RESULTS

### AMPK regulates miRNAs involved in pathways relevant for β-cell function

To identify miRNAs regulated by AMPK in the β cell, we performed miRNA profiling on islets from mice with β-cell–specific deletion of both catalytic subunits of AMPK (βAMPKdKO) *vs.* controls . RNA was extracted from islets from 3 βAMPKdKO and 3 control animals and analyzed by Exiqon using qPCR panels. These allow the simultaneous detection of 752 mature miRNAs. Of those, an average of 376 miRNAs were detected per sample, and 323 were detected in all samples.

**Figure 1.**
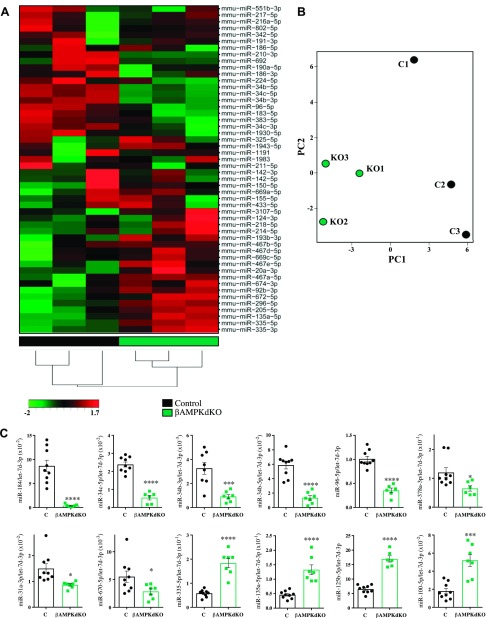
AMPK regulates miRNA expression in pancreatic islets. *A*, *B*) Heat map and unsupervised hierarchical clustering (*A*) and PCA (*B*) on the 3 control (black, C) and 3 βAMPKdKO (green, KO) samples and top 50 miRNAs with the highest sd. *C*) miRNA expression was assessed by RT-qPCR in isolated islets from βAMPKdKO (green) and littermate control (C, black) mice. Each dot represents islets from a single mouse. Data are expressed as relative to the endogenous control let-7d-3p. *P < 0.05, ***P < 0.001, ****P < 0.0001 (Student’s *t* test).

Unsupervised hierarchical clustering (**Fig. 1*A***) and principal component analysis (PCA) (Fig. 1*B*) revealed that, as anticipated, control and βAMPKdKO samples clustered together. With a cutoff value of *P* < 0.05 (Student’s *t* test), 81 miRNAs were found to be differentially expressed in βAMPKdKO *vs.* control islets (Supplemental Table 1). After Benjamini-Hochberg correction (FDR < 0.15), 13 and 9 miRNAs were down- and up-regulated, respectively (Supplemental Table 1 and **Table 2**). We also noticed that the miRNA that was most differentially expressed, miR-184-3p (miR-184), was undetectable in one of the βAMPKdKO samples, and therefore the statistical significance of its change was not evaluated initially (Supplemental Table 1 and Table 2).

**TABLE 2.** miRNAs down- and up-regulated in βAMPKdKO vs. control islets

miRNA	Fold change	FDR
Down-regulated miRNAs		
mmu-miR-184-3p	−33.00	N/A
mmu-miR-34c-5p	−6.13	0.056
mmu-miR-34b-3p	−6.04	0.048
mmu-miR-34b-5p	−4.35	0.048
mmu-miR-96-5p	−2.68	0.128
mmu-miR-378a-3p	−2.16	0.066
mmu-miR-31-3p	−2.05	0.128
mmu-miR-670-5p	−2.02	0.067
mmu-miR-200a-3p	−1.41	0.128
mmu-miR-101a-3p	−1.39	0.141
mmu-miR-30e-5p	−1.34	0.128
mmu-miR-136-5p	−1.34	0.090
mmu-miR-29a-5p	−1.32	0.056
mmu-miR-140-5p	−1.17	0.128
Up-regulated miRNAs		
mmu-miR-9-5p	1.36	0.128
mmu-miR-20a-5p	1.51	0.128
mmu-miR-181a-5p	1.56	0.090
mmu-miR-10b-5p	1.58	0.128
mmu-miR-99a-5p	1.68	0.094
mmu-miR-100-5p	1.88	0.037
mmu-miR-125b-5p	2.20	0.128
mmu-miR-135a-5p	2.57	0.141
mmu-miR-335-5p	4.29	0.057

The average *C*_*q*_ value of all the detected assays was used as normalizer. N/A, not applicable.

**Figure 2.**
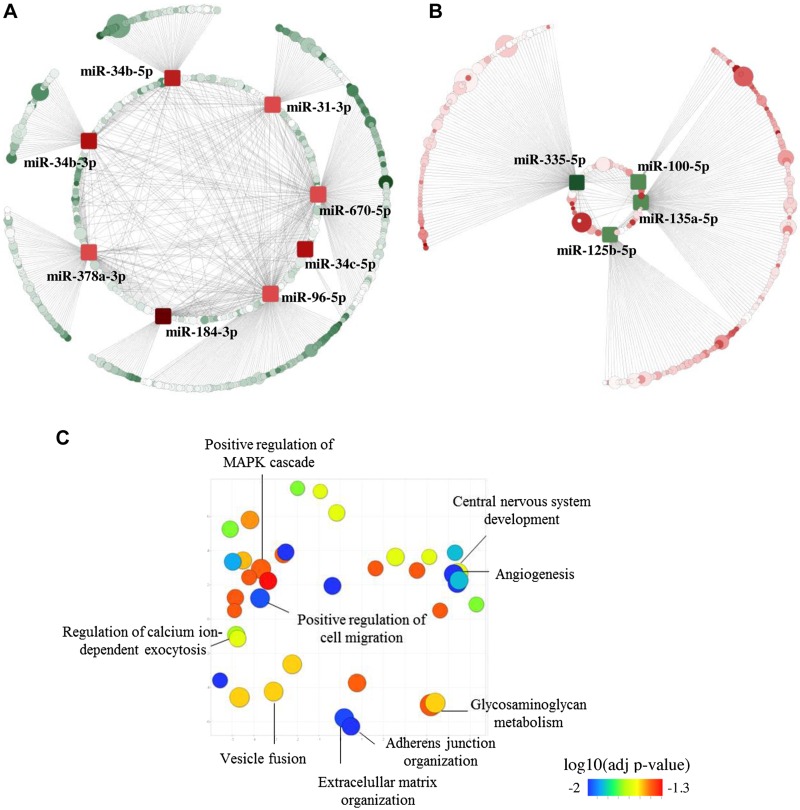
miRNAs regulated by AMPK are involved in pathways important for β-cell function. *A*, *B*) Cytoscape-generated layout of down-regulated (square, red nodes) (*A*) and up-regulated (square, green nodes) (*B*) miRNAs and their predicted targets that are up-regulated (circle, green nodes) (*A*) and down-regulated (circle, red nodes) (*B*), respectively, in βAMPKdKO *vs.* control islets. Node size represents the degree of the fold change (the larger, the stronger). The intensity of the gene node color (circles) indicates the target prediction score according to TargetScan (the darker, the stronger). A full list of miRNA and targets can be found in Supplemental Tables 2 and 3. *C*) REViGO Scatterplot of the enriched GO terms. Up-regulated targets of down-regulated miRNAs (Supplemental Table 2) in βAMPKdKO islets were submitted to EnrichR, which identified enrichment in 37 (Supplemental Table 4) biologic processes (adjusted *P* < 0.05). GO terms along with their adjusted significance values (*P* < 0.05) are represented by circles and are plotted according to semantic similarities with other GO terms. Circle size is proportional to the frequency of the GO term, and circle color represents the adjusted significance value calculated using EnrichR.

To further understand the contribution of the affected miRNAs to AMPK function, we used TargetScan (22) to identify predicted targets of miRNAs, which showed a >2-fold change in βAMPKdKO *vs.* control islets [down-regulated miRNAs (DownmiRs): miR-184, miR-34bc-5p, miR-34b-3p, miR-96-5p, miR-378a-3p, miR-31-3p, miR-670-5p; up-regulated miRNAs: miR-335-5p, miR-135a-5p, miR-125b-5p plus miR-100-5p, with a fold change of 1.88 but a *P*adj <0.05). The effect of β-cell–specific AMPK deletion on the expression of these miRNAs was further validated in islets isolated from a higher number of animals (Fig. 1*C*). Let-7d-3p expression was highly stable (*i.e.*, similar between different samples) according to the panel assays and was therefore used as endogenous control. Comparable results were obtained upon normalization with a second endogenous control, miR-574-3p (data not shown). Because miRNAs act by silencing gene expression, we expect an inverse correlation between the expression of a miRNA and its targets. Thus, using our previously published RNA-seq data (6), we found that 576 predicted DownmiR targets [TargetScan score (cumulative weighted context score) < −0.1] were significantly (*P*adj < 0.1) up-regulated >1.25 fold (UpTargets, **Fig. 2*A*** and Supplemental Table 2] in βAMPKdKO islets, whereas 277 predicted targets of up-regulated miRNAs were down-regulated (DownTargets, Fig. 2*B* and Supplemental Table 3). GO analysis (24, 26) was performed on these predicted targets (UpTargets and DownTargets independently) and revealed enrichment (*P*adj < 0.05) in 37 (Supplemental Table 4) biologic processes for UpTargets. DownTargets were not involved in biological processes that passed the *P*adj threshold, although they might contribute to regulation of ERK1 and ERK2 cascades (*P*adj = 0.084). We submitted our list of enriched GO terms to REVIGO (25), which allows summarization and visualization of long lists of GO terms (Fig. 2*C*).

Predicted targets (UpTargets) of DownmiRs were enriched in pathways important for β-cell function, including adherens junctions and extracellular matrix organization, angiogenesis, vesicle fusion, calcium-dependent exocytosis, and nervous system development (Fig. 2*C* and Supplemental Table 4). We have previously reported (6) that βAMPKdKO islets present with altered secretory function and up-regulation of genes involved in neuronal function. Thus, our results suggest that the identified miRNAs are important contributors to AMPK action in β cells.

### AMPK is both necessary and sufficient for miR-184 expression in mice and humans

As mentioned above, miRNAs down-regulated in βAMPKdKO islets might be involved in several AMPK-regulated biologic processes. Moreover, the most down-regulated miRNA, miR-184, has been demonstrated to be an important regulator of β-cell proliferation during compensation after insulin resistance in mice (14, 17). miR-34b-5p/c-5p (miR-34b/c) was also strongly down-regulated in βAMPKdKO islets. Although the functions of miR-34b/c are unknown in islets, these miRNAs share seed region sequence (and therefore many predicted targets) with the third member of the family, miR-34a, a mediator of compensatory β-cell expansion, lipotoxicity, and apoptosis (12). In addition, miR-96-5p (miR-96) regulates insulin exocytosis in a murine β-cell line (12). Thus, the latter miRNAs were chosen for further study.

**Figure 3.**
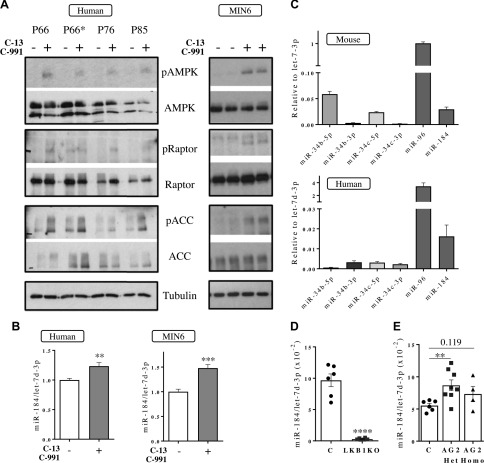
AMPK is both necessary and sufficient for miR-184 expression in mouse and human islets. *A*) Phosphorylation of AMPK at Thr172 (pAMPK) and its targets Raptor (pRaptor) and ACC (pACC) as well as the total protein (AMPK, Raptor, ACC) was assessed by Western blotting using specific antibodies with extracts from human islets (left side panel) or MIN6 cells (right side panel) treated with AMPK activators C13 (20 μM) and C991 (50 μM) for 24 and 14 h, respectively. P66, P76, and P85 are donor identifiers (see details in Table 1). P66 and P66* correspond to the same donor, but P66* corresponds to islets >100 μm. P66, P76, and P85 islets were all <100 μm. *B*–*E*) miR-184 expression was assessed by RT-qPCR in human islets (left hand side, *n* = 5) and MIN6 cells (right hand side, *n* = 3) treated with the AMPK activators as in *A* (*B*), islets from C57BL/6 mice (upper panel, *n* = 8–9) and humans without diabetes (*n* = 7–9) (*C*), islets from βLKB1KO (LKB1KO) and littermate control (C, black) mice (*D*), and islets from R299Q γ2 AMPK knock-in mice [heterozygous (AG2 Het) or homozygous (AG2 Homo) for the mutant allele] and littermate controls (C) (*E*). In *D* and *E*, each dot represents islets from a single mouse. Data are expressed as relative to the endogenous control let-7d-3p. In panel *B*, data are fold-change *vs.* the untreated sample. **P < 0.01, ***P < 0.001, ****P < 0.0001 (Student’s *t* test).

To determine if forced activation of AMPK induces the expression of these miRNAs and if this regulatory mechanism is conserved in human islets, we activated AMPK pharmacologically using two synergistic small, highly specific AMPK activators, C991 (20) and C13 (19). As expected, treatment of human islets with the activators (20 μM C13 plus 50 μM C991) for 24 h resulted in increased phosphorylation of AMPK and its well-characterized targets ACC and Raptor (**Fig. 3*A***). Concomitantly, miR-184 was significantly up-regulated (Fig. 3*B*), although no significant changes were observed in the expression of miR-34b/c, miR-34b-3p, and miR-96 (data not shown). Indeed, miR-34b/c levels, which are low in mouse islets, were barely detectable in human islets (Fig. 3*C*), suggesting that the role of these miRNAs in β cells, if any, might not be conserved in humans. Correspondingly, overnight treatment of MIN6 β cells with AMPK activators increased miR-184 expression (Fig. 1*A*, *B*).

Further supporting the importance of AMPK in controlling miR-184 expression, islets with β-cell–specific deletion of LKB1 (6) displayed reduced miR-184 levels (Fig. 3*E*). Moreover, islets from mice bearing an activating mutation of AMPK γ2 [R299Q γ2 AMPK knock-in mice (3)] showed elevated miR-184 levels (Fig. 1*F*).

Together, our results indicate that AMPK is both necessary and sufficient for miR-184 expression in mouse and human β cells.

### miR-184 expression is sex dependent

**Figure 4.**
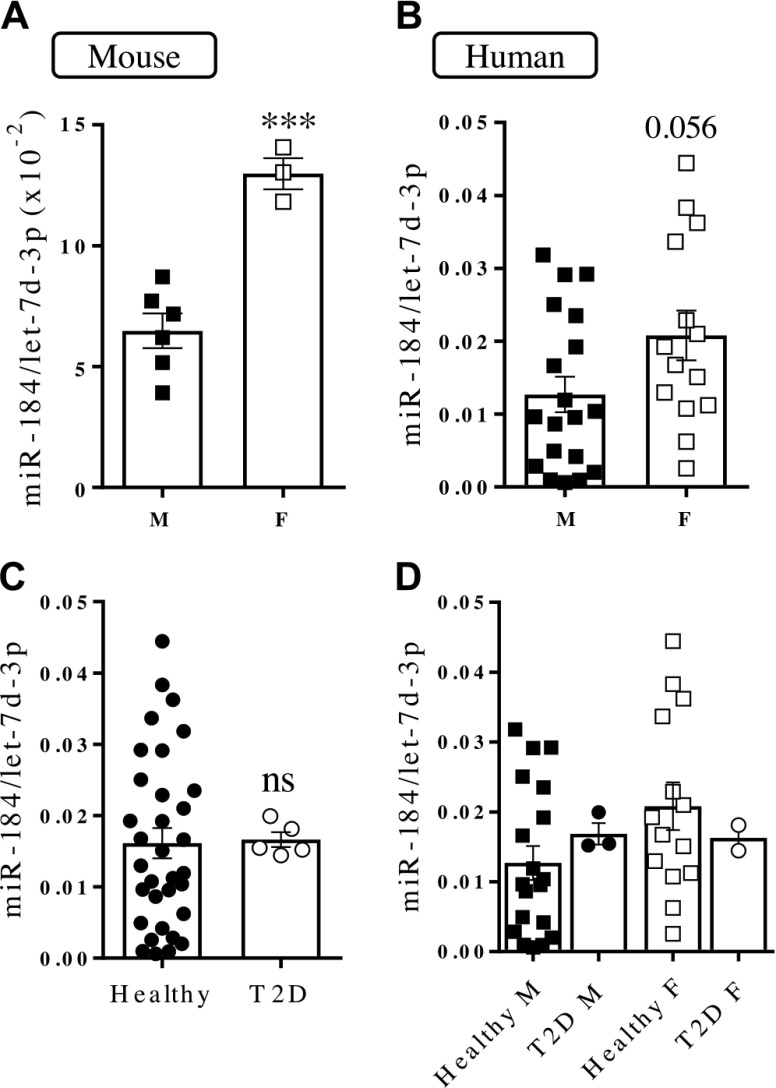
miR-184 expression is sex dependent. miRNA expression was assessed by RT-qPCR in isolated islets from male (M) and female (F) control mice (*A*), human islets from male and female donors (*B*), human islets from healthy donors and donors with T2D (*C*), and human islets from healthy male and female donors and male and female donors with T2D (*D*). Each dot represents islets from a single mouse (*A*) or human donor (*B*–*D*). Data are expressed as relative to the endogenous control let-7d-3p. ns, not significant. ***P < 0.001 (Student’s *t* test).

While performing the experiments described above, we noticed that islets from control animals contained highly variable levels of miR-184. Plotting of the values shown in Fig. 1*C* according to sex revealed that islets from female mice express significantly higher levels (>2 fold, *P* < 0.001) of miR-184 *vs.* male islets (**Fig. 4*A***). To determine whether this sex dependence is conserved in humans, we assessed miR-184 expression in islets from 19 male and 14 female nondiabetic donors (Table 1). Islets from female donors expressed higher levels of miR-184 (1.6 fold, *P* = 0.056, power of ∼61% probability of rejecting a null hypothesis) (Fig. 4*B*). Levels of other miRNAs were similar in male and female islets (data not shown).

It has been reported that miR-184 is down-regulated in islets from T2D donors (17). In our study, miR-184 expression was similar in islets from donors with T2D and healthy donors (Fig. 4*C*). miR-184 levels tended to be lower in female patients with T2D *vs.* control subjects (Fig. 4*C*, *D*), whereas this trend was not observed in male donors.

miR-184 expression did not correlate with age or body mass index from the donors, which was similar within the compared groups (Supplemental Fig. 1 and Table 1).

### AMPK mediates the glucose-dependent regulation of miR-184 in mouse islets

Tattikota *et al.* (18) have previously reported that miR-184 is regulated according to glucose metabolism both *in vivo* and *in vitro*. These researchers found that miR-184 is slightly down-regulated in mouse islets cultured at high glucose concentrations and is strongly increased in islets from mice starved for >30 h or fed a low-sugar/ketogenic diet. Nevertheless, the mechanisms underlying this glucose dependency of miR-184 expression have remained unexplored. Given the strong effect of AMPK on miR-184 expression, we hypothesized that AMPK mediates the effects of glucose on miR-184.

**Figure 5.**
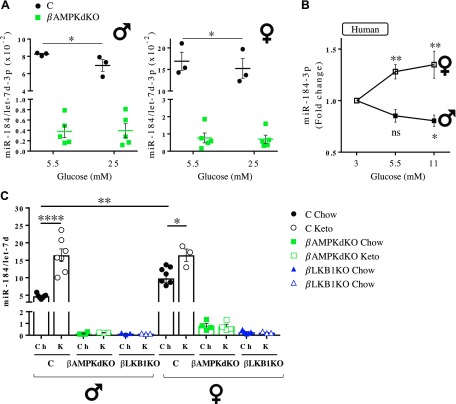
AMPK mediates glucose-dependent regulation of miR-184. miRNA expression was assessed by RT-qPCR in isolated islets from βAMPKdKO (green) and control (C, black) male (left side) and female (right side) mice cultured with 5.5 or 25 mM glucose for 48 h (*A*); male and female human donors cultured with 3, 5.5, or 11 mM glucose for 48 h (*B*); and βAMPKdKO (green), βLKB1KO (blue), and control (C, black) male and female mice fed chow (Ch) or a ketogenic (K) diet for 28 d (*C*). Ns, not significant. *P < 0.05, **P < 0.01, ****P < 0.0001 [Student’s *t* test (*A*, *C*) or 2-way repeated-measures ANOVA and Fisher least significance difference test (*B*)].

Treatment of isolated mouse islets with increasing concentrations of glucose for 48 h led to the dephosphorylation of AMPK at Thr-172 as well as its targets Raptor and ACC (Supplemental Fig. 2*A*). In inverse correlation with AMPK activation and consistent with previously published results (18), miR-184 expression was slightly but significantly reduced in control islets incubated at high glucose (**Fig. 5*A***). In contrast, miR-184 expression remained unchanged in βAMPKdKO islets (Fig. 5*A*), suggesting that AMPK is required for the glucose-dependent regulation of miR-184 expression *in vitro*. Similarly, treatment of human islets with high glucose led to dephosphorylation of AMPK and Raptor (Supplemental Fig. 2*B*), although ACC phosphorylation remained unchanged (Supplemental Fig. 2*B*). Surprisingly, whereas miR-184 expression was decreased in islets from male donors treated with increasing glucose concentrations, miR-184 was consistently increased in islets from female donors (Fig. 5*B*). *TXNIP*, which is potently up-regulated at high glucose (27), was clearly increased (Supplemental Fig. 2*C*) in both male and female islets.

Next, we studied the AMPK dependence of the effects of glucose on miR-184 expression *in vivo* using conditions similar to those of Tattikota *et al.* (18) in our β-cell–specific βAMPKdKO and βLKB1KO mouse models. First, control and βLKB1KO animals were held without food overnight (16 h). Contrary to published results (18), miR-184 expression remained unchanged in unfed *vs.* fed control animals (Supplemental Fig. 3*A*). Of note, 16 h without food did not cause obvious changes in AMPK activation (Supplemental Fig. 3*B*, *C*).

Subsequently, we fed control and knockout animals a ketogenic (low-carbohydrate) diet that, as anticipated, resulted in an early loss of weight (Supplemental Fig. 4*A*) and lowered glycemia (Supplemental Fig. 4*B*). In agreement with Tattikota *et al*. (18), in our study miR-184 was sharply increased in control mice fed a ketogenic diet (Fig. 5*C*). By contrast, miR-184 expression in islets from βAMPKdKO and βLKB1KO mice remained unchanged (Fig. 5*C*), confirming that AMPK is required for glucose-mediated regulation of islet miR-184 *in vivo*. The fold increase induced by the ketogenic diet was much higher in male (3.5-fold) than in female islets (1.6-fold), with sex-dependent differences in miR-184 expression eliminated on a ketogenic diet.

**Figure 6.**
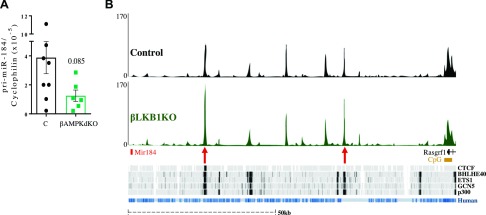
AMPK may mediate miR-184 expression at the transcriptional level. *A*) Pri-miR-184 expression was measured by RT-qPCR in isolated islets from βAMPKdKO (green) and littermate control (C, black) mice. *B*) Integration of ATAC-seq enrichment profiles with ENCODE CTCF and transcription factors ChIP-seq datasets. Sequencing tracks for the ∼100-kb genomic region upstream miR-184 show 2 enriched ATAC-seq peaks (red arrows) in βLKB1KO (green) *vs.* control (black) islets. CTCF ChIP-seq profiles in liver show enrichment of this factor within the most proximal ATAC-seq differential peak. Binding enrichment of other transcriptional regulators (CH12 cells) is observed at both differential ATAC-seq peaks. Alignment with the human DNA track is presented in blue.

MiRNAs are transcribed as primary RNAs of variable length that are processed in a two-step manner that requires several enzymes and can be regulated at different levels (7). Expression of miR-184 primary transcript was lower in βAMPKdKO than control islets (**Fig. 6*A***), suggesting that AMPK regulates miR-184 expression at the transcriptional level. The exact sequence of pri-miR-184 and its promoter and regulatory sequences have not been fully annotated and/or experimentally validated. Nevertheless, the *MIR184* transcription start site might be located up to ∼78 kb upstream of the miRNA loop (28). Our unpublished assay for transposase-accessible chromatin and sequencing (ATAC-seq) data (unpublished results) demonstrated a significant increase in chromatin accessibility in βLKB1KO islets in two regions located ∼25 kb (1.3-fold, *P* = 0.0074, *P*adj = 0.08) and ∼72 kb (1.2-fold, *P* = 0.0456, *P*adj = 0.23) upstream of miR-184 (Fig. 6*B*). ENCODE ChIP-seq datasets from liver and CH12 cells show that the insulator CCCTC-binding factor (*CTCF*) could bind at least the most proximal region (Fig. 6*B*), conserved in human islets, which could result in repression of miR-184. Other factors, such as BHLHE40, ETS1, GCN5, or p300, may bind the most distal region (Fig. 6*B*).

## DISCUSSION

### Contribution of miRNAs to AMPK signaling in β cells

The role of AMPK in the β cell has been strongly debated in recent years [reviewed by Fu *et* *al*. (29)]. Genetic studies (3, 4, 6) demonstrate that, paradoxically, AMPK depletion results in increased glucose-stimulated insulin secretion in isolated islets as well as strongly impaired insulin secretion *in vivo*. These defects occurred in parallel with changes in the expression of thousands of mRNAs that suggested impaired β-cell identity (6). Here, we demonstrate that AMPK deletion produces changes in the levels of at least 23 islet miRNAs, which are predicted to target ∼850 AMPK-regulated mRNAs. Although a limitation of this study is that those miRNA–target interactions have not been experimentally validated, GO analysis of these targets reveals enrichment in biologic processes that point to miRNAs as important contributors to AMPK action.

On the top of the list of enriched biologic pathways, we found angiogenesis, positive regulation of cell migration, and adherens junction organization. Angiogenesis is essential for islet development and central to the capacity of the mature islets to sense and fulfill metabolic demands (30). Likewise, the cell migration machinery and adherens junctions have been proposed to influence β-cell differentiation, survival, and insulin secretion (17, 31).

#### Impaired β-cell identity

Suggesting impaired identity, neuronal genes were up-regulated in βAMPKdKO islets (6). MiRNAs have been described as important regulators of cell fate and efficient fine-tuners of gene expression (32, 33). Deletion of Dicer (essential for miRNA maturation) in Ngn3^+^ progenitors results in dysfunctional adult β cells with up-regulation of several neuronal genes, such as *Rest* and *Phox2a/b* (9). *Rest* is up-regulated in βAMPKdKO islets (∼1.5-fold) (6) and is a predicted target of the down-regulated (∼2-fold) miR-670-5p and miR-200a-3p (1.4-fold). Several other genes up-regulated in βAMPKdKO islets and predicted targets of DownmiRs (UpTargets; Supplemental Table 2) are involved in nervous system development (Supplemental Table 4), although their role in β cells is unknown.

Other miRNA–target pathways that may contribute to the loss of β-cell identity after AMPK deletion are the up-regulated *Ldha*, a validated target of miR-34a/b/c in other cell types (34, 35), and *Sox6*, which can lower Pdx-1 activity to attenuate glucose stimulated insulin secretion (36) and is targeted by miR-96 in hepatocellular carcinoma (37).

#### Insulin secretion

miRNAs might contribute to the defects observed in secretion by regulating components of the vesicle fusion machinery, such synaptotagmins and *Doc2b* (38, 39), which are predicted targets of the DownmiRs miR-31-3p, miR-378a-3p, miR-184, miR-34b-5p, and miR-670-5p.

Targets of miRNAs that are negatively regulated by AMPK and that might affect insulin synthesis and exocytosis include tyrosine hydroxylase (*Th*), a predicted target of miR-335-5p that is required for β-cell development and promotes insulin synthesis (40). *MAPT* has been found to increase β-cell proliferation at the expense of insulin secretion (41) and is a predicted target of miR-135a-3p and miR-125b. Moreover, the Akt-targeted Rab GTPase–activating protein *TBC1D4*, which regulates IBMX- and glucose-stimulated insulin secretion by EndoC-βH1 and MIN6 cells, respectively (42), is a putative target of miR-135a and miR-125b. MiR-135a also has binding sites in *Gcgr* 3′UTR, whose depletion causes impaired β-cell responses to glucose and other secretagogues (43). The action of these miRNAs may therefore contribute to the defects in insulin secretion observed in βAMPKdKO animals (6).

### Role of miR-184 in AMPK function

The function of miR-184 in the β cell has been intensively studied by Tattikota *et al.* (17). These researchers first overexpressed this miRNA in MIN6 cells and identified several down-regulated genes, including *Ago2*, at the RNA and protein level. Ago2 is an important component of miRISC, which is necessary to mediate miRNA-dependent repression. Tattikota *et al*. (17) subsequently generated a constitutive miR-184 knockout model in which *Ago2* expression was slightly but significantly increased and which presented with reduced fasted glucose but increased plasma insulin levels and β-cell mass. Conversely, the researchers overexpressed miR-184 specifically in β cells, and this resulted in reduced *Ago2* expression. In contrast, overexpression of *Ago2* in the presence of miR-184 restored normal glucose control (17). Tattikota *et al*. (17) concluded that a decrease in miR-184 expression was required for compensatory β-cell proliferation through up-regulation of *Ago2*, whereas the effect of miR-184 on insulin secretion might be mediated by other targets, such as the mitochondrial glutamate transporter *Slc25a22*, which has been proposed to play a positive role in glucose-stimulated insulin secretion (44).

Neither βAMPKdKO nor βLKB1KO islets display changes in *Ago2* expression, as determined by our previously published RNA sequencing data (6) and further validated at the RNA level in >6 animals by RT-qPCR and at the protein level by Western blot (data not shown). This is consistent with the opposite effects in β-cell mass in our βAMPKdKO animals *vs.* the miR-184 knockouts generated by Tattikota (17). The mouse and human Ago2 3′UTRs are exceptionally long (>11 kb) and therefore contain binding sites for multiple miRNAs. These include four of those most up-regulated in βAMPKdKO islets: miR-135a, miR-125b, miR-100, and miR-99a, with the last two being experimentally validated in other cell types (45, 46). These up-regulated miRNAs might compensate for the lack of miR-184 in the absence of AMPK, avoiding a dynamic and energy-dependent sensor affecting the expression of such a pleotropic protein as AGO2.

On the other hand, βAMPKdKO islets displayed a significant increase in *Slc25a22* expression *vs.* controls. Thus, miR-184 could, through this target, contribute to the increased insulin secretion observed in isolated βAMPKdKO islets. Interestingly, *Slc25a22* has previously been proposed to mediate miR-184 effects in mitochondria morphology and number (18).

### Role of AMPK in the regulation of miR-184 expression

miR-184 is strongly down-regulated in islets from prediabetic and diabetic *db/db* and *ob/ob* mice and mice fed a high-fat diet (14, 17). A negative correlation has also been observed between the expression of this miRNA and the insulin secretion index of human islets (47). In subsequent studies, Tattikota *et al.* (18) reported that miR-184 is regulated by glucose in mice and in *Drosophila melanogaster*. *In vivo*, both a ketogenic diet (with virtually no calories deriving from carbohydrates) or starvation for 25–30 h resulted in islet miR-184 up-regulation. Nevertheless, none of these studies investigated the mechanisms underlying the regulation of this miRNA.

We demonstrate here that depletion of AMPK selectively from the β cell strongly impairs miR-184 expression and that this also occurs after depletion of the AMPK upstream kinase LKB1. Moreover, a forced increase in AMPK activity resulted in miR-184 up-regulation in a murine cell line (MIN6), in mouse islets (AMPKγ2 animals), and, most importantly, in human islets.

#### miR-184 is differentially expressed according to sex

The current study revealed that miR-184 is differentially expressed in islets from male *vs.* female mice, with a similar strong tendency (*P* = 0.056) observed in human islets. Sexual dimorphism in the expression of miR-184 has also previously been observed in ticks (*Rhipicephalus haemaphysaloides*) (48) and *Drosophila* (49).

The studies described in refs 17, 18 were apparently done using mice of the same sex and human islets from both male and female donors, which might have masked the existence of sexual dimorphism in the expression of miR-184. Conversely, this sexual dimorphism may explain, at least in part, the discrepancies between our study and that of Tattikota *et al.* (17), who reported lower miR-184 expression in T2D islets *vs.* subjects without diabetes. Even though our study is limited in this regard by the low number of T2D samples, two additional independent studies (50, 51) found no differences in the levels of miR-184 in diabetic *vs.* nondiabetic islets. Further study is needed to reinforce the findings reported here.

In general, the incidence of T2D is similar in men and women, although differences in glucose metabolism and insulin action have been previously described. A strong gender bias has also been observed in animal models of T2D (52). Any role that the pancreatic β cell plays in sexual dimorphism is unknown. Recently, Hall *et al.* (53) found sex-dependent differences in the DNA methylation pattern in the X-chromosome and in CpG sites of autosomal chromosomes in human islets. Even though the β-cell number was identical between male and female subjects, female islets displayed higher insulin secretion (stimulation index). Changes in DNA methylation were associated with differences in the expression of certain miRNAs and protein-coding genes, such as *NKAP*, which inhibits GSIS through NF-κB activation and 10 genes associated with T2D by genome-wide association studies. Nevertheless, neither the methylation status of the miR-184 promoter nor the expression levels of this miRNA was evaluated.

AMPK was previously reported to show a sex bias in terms of its activation in response to metabolic stimuli in liver and adipose tissue, where estrogens affect lipid accumulation by activating AMPK (54). Sex-biased AMPK activation, which may contribute to differential miR-184 expression in islets, has not previously been described. Providing a possible mechanistic underpinning of this observation, 17β-estradiol transiently stimulates β-cell AMPK phosphorylation to an extent close to that seen in response to low glucose (55). Another exciting hypothesis is the existence of a regulatory serotonin–AMPK–miR-184 pathway. Thus, in *Caenorhabditis* *elegans*, AMPK links serotonergic signaling with glutamate release (56), whereas in the Aplysia nervous system serotonin down-regulates miR-184 (57). Expression of serotonin receptors and serotonin synthesis may also differ according to sex (58) and are enhanced in islets during pregnancy (59).

#### Role of AMPK in regulation of miR-184 by glucose

We demonstrate that AMPK is required for the glucose-mediated regulation of murine miR-184 expression because culturing islets at different glucose concentrations or feeding the mice a ketogenic diet had no effect on miR-184 expression in the absence of AMPK. Feeding a ketogenic diet has been demonstrated to increase AMPK activity in liver and muscle (60). In our study, miR-184 expression remained unchanged after 16 h without food, in contrast with results obtained by Tattikota *et al.* (18). The mice used by Tattikota *et al*. (18) had the same genetic background as those used in this study but were starved for a longer period (30 h).

Paradoxically, culture of human islets at different glucose concentrations exerted opposing effects on miR-184 according to sex. Increasing glucose concentrations reduced miR-184 in male human islets [as observed in both male and female mice islets (Fig. 5*B*)]. The same treatment increased the expression of the miRNA in female islets, whereas AMPK activity was increased in both male and female islets (Supplemental Fig. 2). The underlying causes of these differences remain to be studied.

Although we have not studied the role of AMPK in the control of miR-184 in animals maintained on a high-fat diet, AMPK phosphorylation is greatly reduced in islets from *db/db* mice at low glucose concentration *vs.* control (db/+) mice (61) as well as in those from diet-induced obesity (62). We therefore hypothesize that AMPK might underlie the changes in miR-184—and perhaps other miRNAs—expression observed by Nesca *et al*. (14) and Tattikota *et al*. (17).

While the present manuscript was under revision, Gendron and Pletcher (63) reported that miR-184 was subject to dietary control and contributed to longevity in *Drosophila*. It is therefore tempting to speculate that this miRNA may contribute to the previously elucidated role of AMPK (64) in controlling lifespan in this species.

### Regulation of miR-184 transcription

Our data suggest that the action of AMPK on miR-184 occurs at the transcriptional level. Correspondingly, our unpublished ATAC-seq data demonstrate a significant increase in chromatin accessibility in βLKB1KO islets in 2 regions located ∼25 and ∼72 kb upstream miR-184. Data from ENCODE suggest that CTCF can bind to the proximal region, whereas MeCP2 binding has been mapped to the most distal location (28), which could contribute to repression of miR-184 in βLKB1 islets. In mouse brain, MeCP2 remains bound to the maternal miR-184 allele (28), possibly contributing to the imprinting of miR-184 in this tissue, which remains to be studied in islets. Future studies will aim to validate these interactions in mouse and human islets and to determine if or how AMPK and LKB1 affect the activity of these transcriptional regulators.

## CONCLUSIONS

Our work identified an upstream regulator of miR-184 and provided a mechanistic link between altered substrate metabolism and the expression of any miRNA in β cells. We thus describe a novel signal transduction pathway whereby glucose, acting *via* AMPK, controls the expression of a critical noncoding RNA and, consequently, genes involved in β-cell identity and function. Dysregulation of this pathway by hyperglycemia or other metabolic changes may thus contribute to the development of T2D in susceptible subjects.

## Supplementary Material

This article includes supplemental data. Please visit *http://www.fasebj.org* to obtain this information.

## Abbreviations

βAMPKdKO: β-cell–specific AMPK double-knockout
βLKB1KO: β-cell–specific liver kinase B1 knockout
ACC: acetyl-CoA-carboxylase
ATAC-seq: assay for transposase-accessible chromatin and sequencing
C13: compound 13
C991: compound 991
DownmiR: down-regulated microRNA
FBS: fetal bovine serum
FDR: false discovery rate
GO: gene ontology
LKB1: liver kinase B1
miRNA: microRNA
PCA: principal component analysis
qPCR: quantitative PCR
T2D: type 2 diabetes
