# Supplementary material for: MiR-184 expression is regulated by AMPK in pancreatic islets
Source: FASEB J. 2018 Jan 8;32(5):2587–600. doi: 10.1096/fj.201701100R (PMC6207280; doi:10.1096/fj.201701100R)
Supplement: Supplementary file 2 [file fj.201701100R.sf1.pdf]

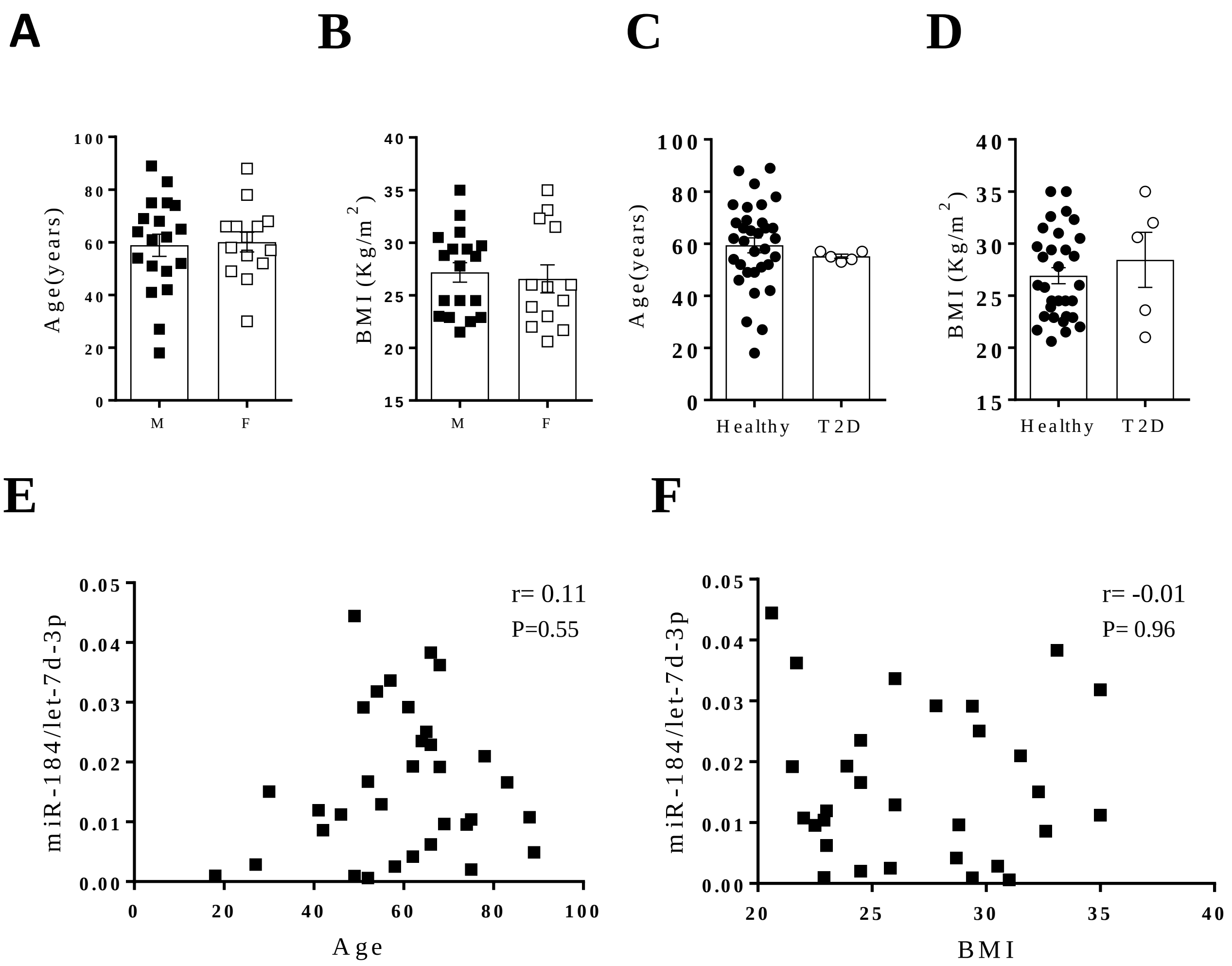

Supplemental Figure 1. MiR-184 expression in human islets is not dependent on age or BMI of the donors. Age **(A, C)** and BMI **(B, D)** is similar within male (M) and female (F) and healthy (Healthy) and diabetic (T2D) donors. MiRNA expression was assessed by RT-qPCR in isolated islets from different healthy donors and plotted according to age **(E)** and BMI **(F).** Pearson correlation coefficients (r) and their corresponding p values (P) are indicated. Each dot represents islets from a single human donor. Data are expressed as relative to the endogenous control let-7d-3p.
