# Supplementary material for: MiR-184 expression is regulated by AMPK in pancreatic islets
Source: FASEB J. 2018 Jan 8;32(5):2587–600. doi: 10.1096/fj.201701100R (PMC6207280; doi:10.1096/fj.201701100R)
Supplement: Supplementary file 3 [file fj.201701100R.sf2.pdf]

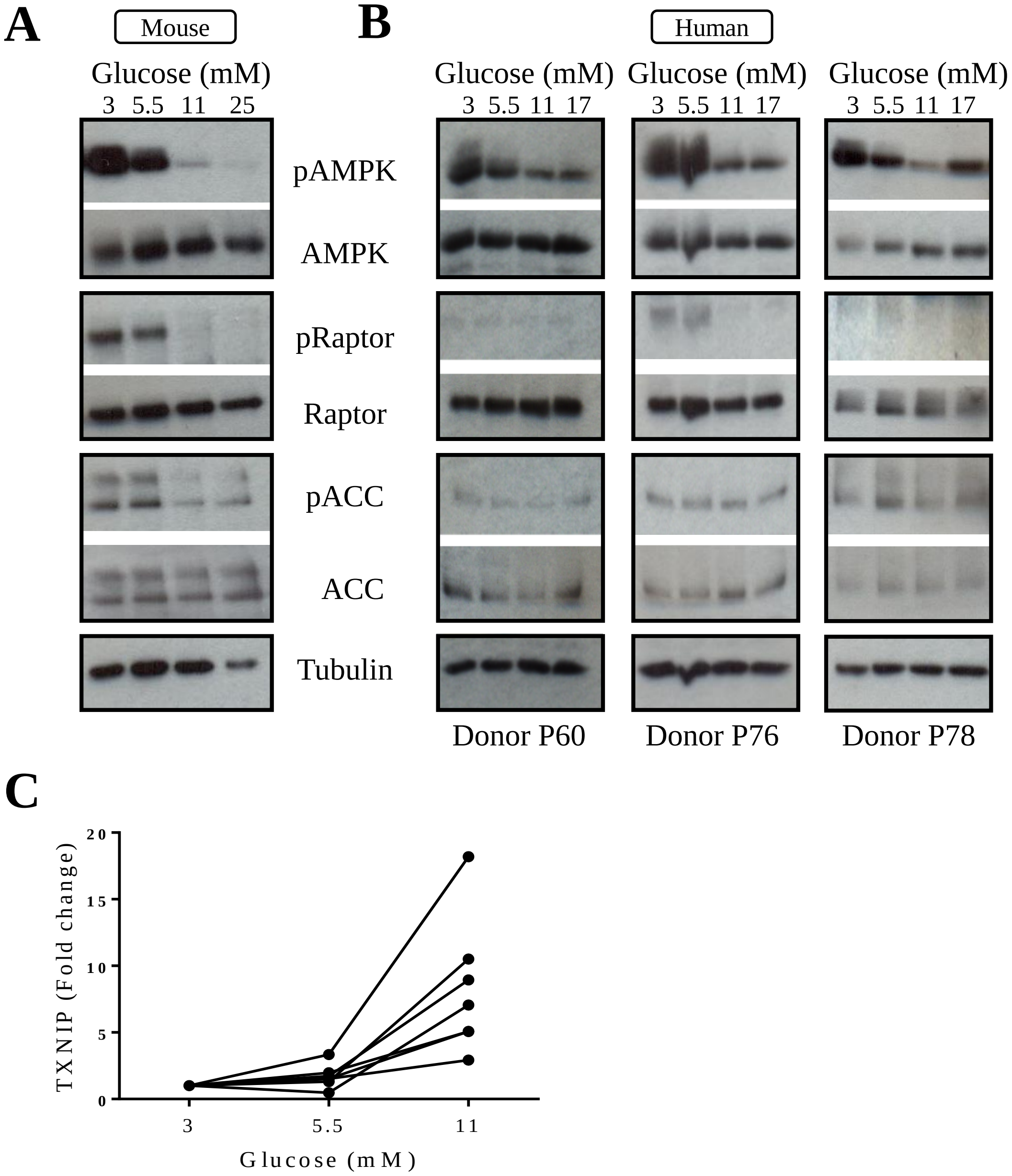

Supplemental Figure 2. Increasing glucose concentration reduces AMPK activity in mouse and human islets. Phosphorylation of AMPK at Thr172 (pAMPK) and of its targets Raptor (pRaptor) and ACC (pACC) as well as total protein (AMPK, Raptor, ACC) assessed by Western (immuno-) blotting using specific antibodies with extracts from **A)** mouse islets and **B)** human islets from three different donors cultured at the indicated glucose concentration for 48h. **C)** *TXNIP* expression was assessed in human islets cultured at the indicated glucose concentration for 48h. Data were normalized by the expression of endogenous *Ppia* and are expressed as fold-change vs. the 3mM glucose sample. Each line represents islets from a different donor and correspond to those used in Figure 5B.
