# Supplementary material for: MiR-184 expression is regulated by AMPK in pancreatic islets
Source: FASEB J. 2018 Jan 8;32(5):2587–600. doi: 10.1096/fj.201701100R (PMC6207280; doi:10.1096/fj.201701100R)
Supplement: Supplementary file 4 [file fj.201701100R.sf3.pdf]

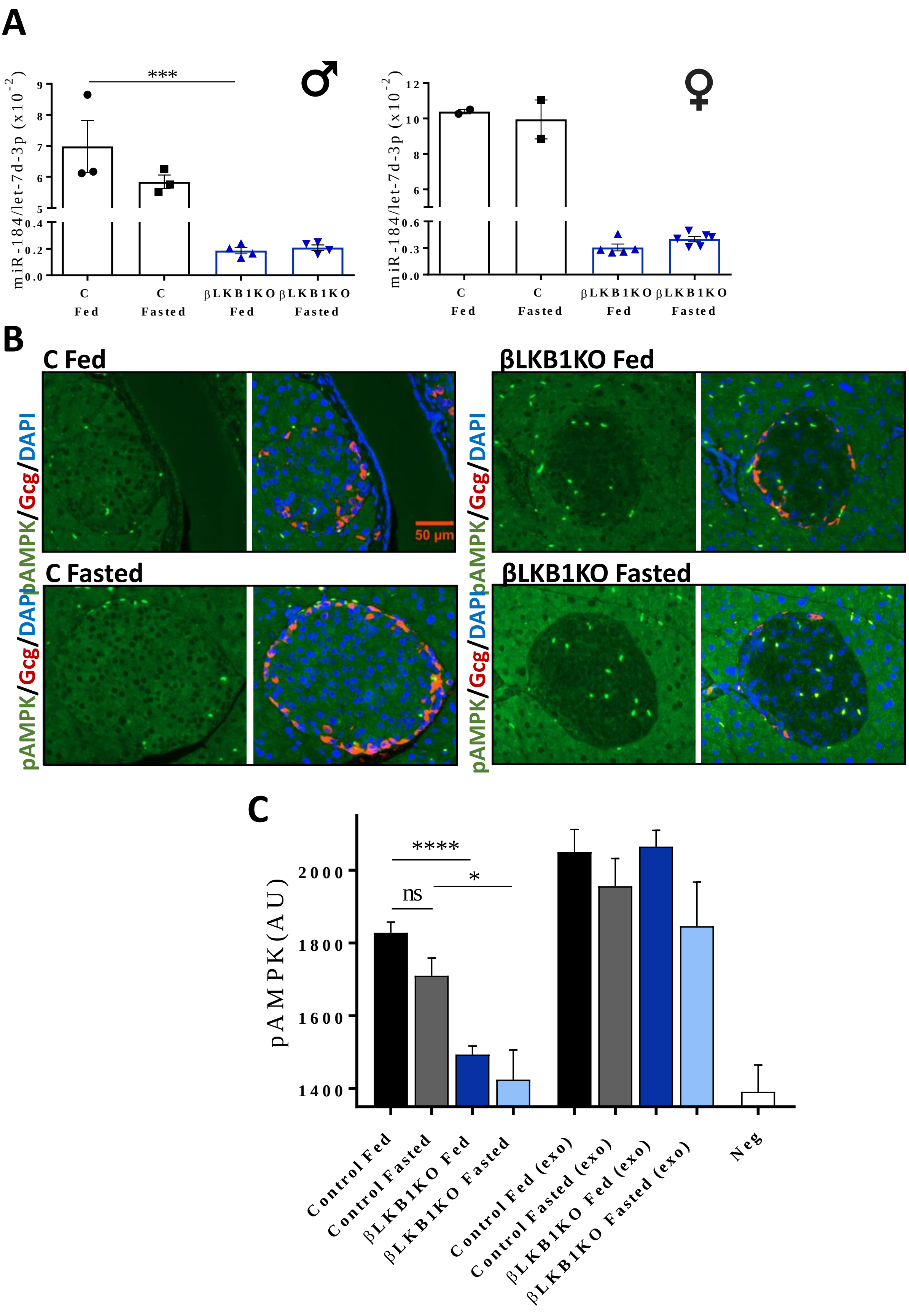

Supplemental Figure 3. 16h fasting does not affect miR-184 nor AMPK phosphorylation in β-cells. A) MiRNA expression was assessed by RT-qPCR in isolated islets from βLKB1KO (blue) and control (C, black) male and female mice fed or fasted during 16h. ***p <0.001, ****p <0.0001, Student t test. **B, C)** Pancreata from βLKB1KO (blue) and control (C, black) mice fed or fasted 16h were fixed and subjected to immunocytochemical analysis for phosphor-AMPK Thr172 and glucagon. Glucagon staining delimits the β-cell surface. Co-staining with insulin was avoided to prevent the bleed-through of the signal masking that from phosphor-AMPK, which is faint in this tissue. Representative slides are shown in **(B)**. **C)** Image software was used to quantify fluorescence intensity of phospho-AMPK staining in the β-cell area (first four bars) and surrounding exocrine tissue (exo). Neg= intensity of the signal in the absence of phospho-AMPK antibody. N=43-130 islets from at least two slides separated by > 500µm, from 2-3 different mice.
