# Supplementary material for: MiR-184 expression is regulated by AMPK in pancreatic islets
Source: FASEB J. 2018 Jan 8;32(5):2587–600. doi: 10.1096/fj.201701100R (PMC6207280; doi:10.1096/fj.201701100R)
Supplement: Supplementary file 5 [file fj.201701100R.sf4.pdf]

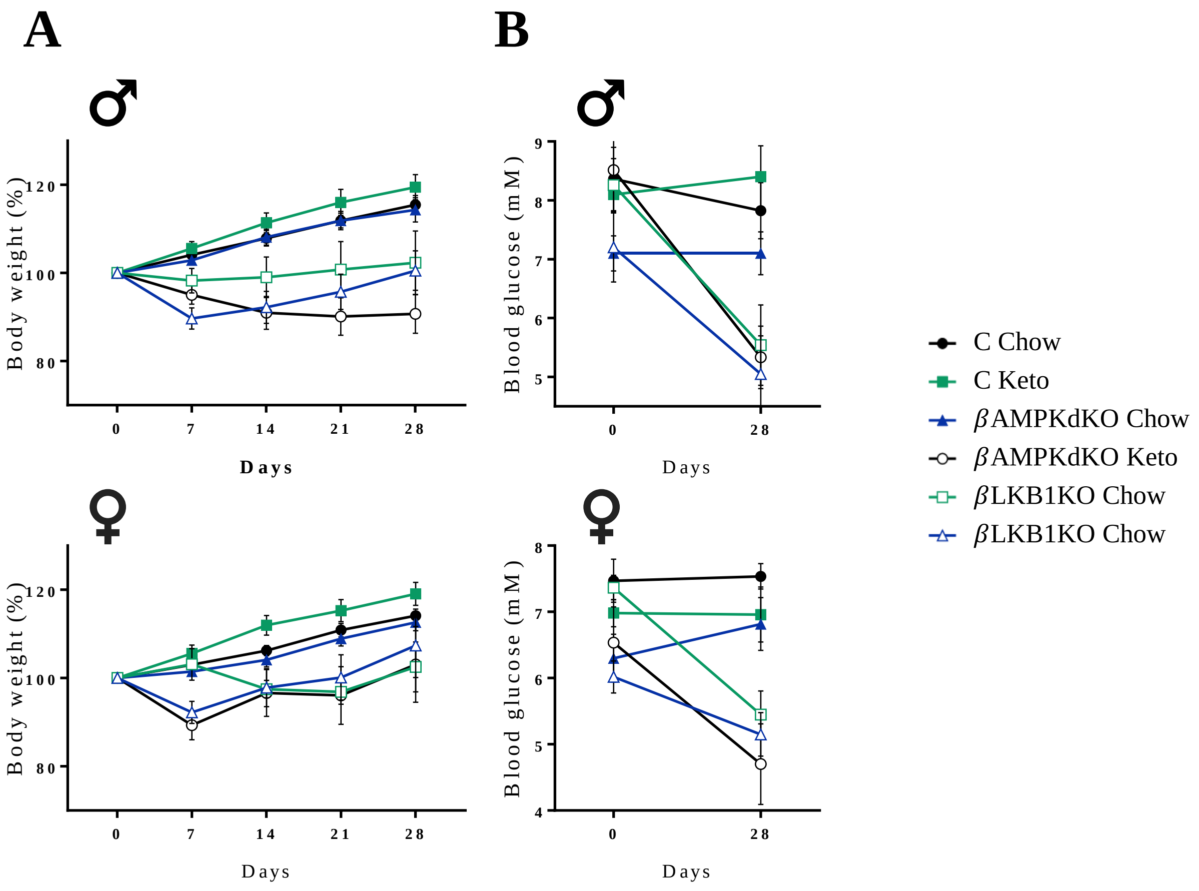

Supplemental Figure 4. Mice fed a ketogenic diet display reduced weight and glycaemia A) Body weight and **B)** randomly-fed glycaemia of βAMPKdKO (green), βLKB1KO (blue) and control (C, black) male and female mice fed a chow (Chow-full symbols) or a ketogenic (Keto-empty symbols) diet for 28 days.
